# A Rare Case of Locally Contracted Cutaneous Corynebacterium diphtheriae

**DOI:** 10.7759/cureus.40854

**Published:** 2023-06-23

**Authors:** Marc Trubin, Sarah E Eichinger, Annette Abraham, Shivanjali Shankaran

**Affiliations:** 1 Internal Medicine, Rush University Medical Center, Chicago, USA; 2 Internal Medicine/Primary Care, Rush University Medical Center/John H. Stroger, Jr. Hospital of Cook County, Chicago, USA; 3 Infectious Disease, Rush University Medical Center, Chicago, USA

**Keywords:** intravenous drug user, vaccine-preventable disease, vaccine, diphtheria, cutaneous diphtheria

## Abstract

A 32-year-old man with a history of intravenous heroin use and housing instability presented with three years of worsening left forearm and wrist “infection,” which had progressed over the past few months with worsening purulence, pain, and deformity. In the emergency department, he was afebrile with stable vitals. Superficial cultures drawn demonstrated polymicrobial growth, including heavy growth of Corynebacterium diphtheriae. He was treated with vancomycin and then IV penicillin to complete 10 days of therapy. Given the uncharacteristic appearance of the lesion, a biopsy was recommended, but the patient left against medical advice. Later, the diphtheria isolate was identified as C. diphtheriae var. mitis by the Centers for Disease Control and Prevention (CDC). This describes an atypical case of cutaneous diphtheria, a disease that is infrequently seen in the United States due to the high prevalence of routine vaccination.

## Introduction

Corynebacterium diphtheriae is a Gram-positive bacillus, which can cause mortality and significant morbidity. There are two predominant clinical syndromes associated with C. diphtheriae infection: respiratory and cutaneous. Cutaneous diphtheria generally presents with chronic, non-healing sores or shallow ulcers with a gray membrane.

C. diphtheriae has largely been eradicated in the developed world due to high vaccination rates. It has been isolated from skin sources recently in impoverished populations, intravenous drug users, and travelers returning to the USA from endemic countries [1,2]. While toxigenic strains are more likely to cause a systemic illness syndrome, non-toxigenic strains can also cause disease [3].

## Case presentation

A 32-year-old man with a history of intravenous heroin use and housing instability presented with three years of worsening left forearm and wrist “infection.” Over the past several months, he had ongoing purulent drainage, pain, and deformity of the left upper extremity.

**Figure 1:**
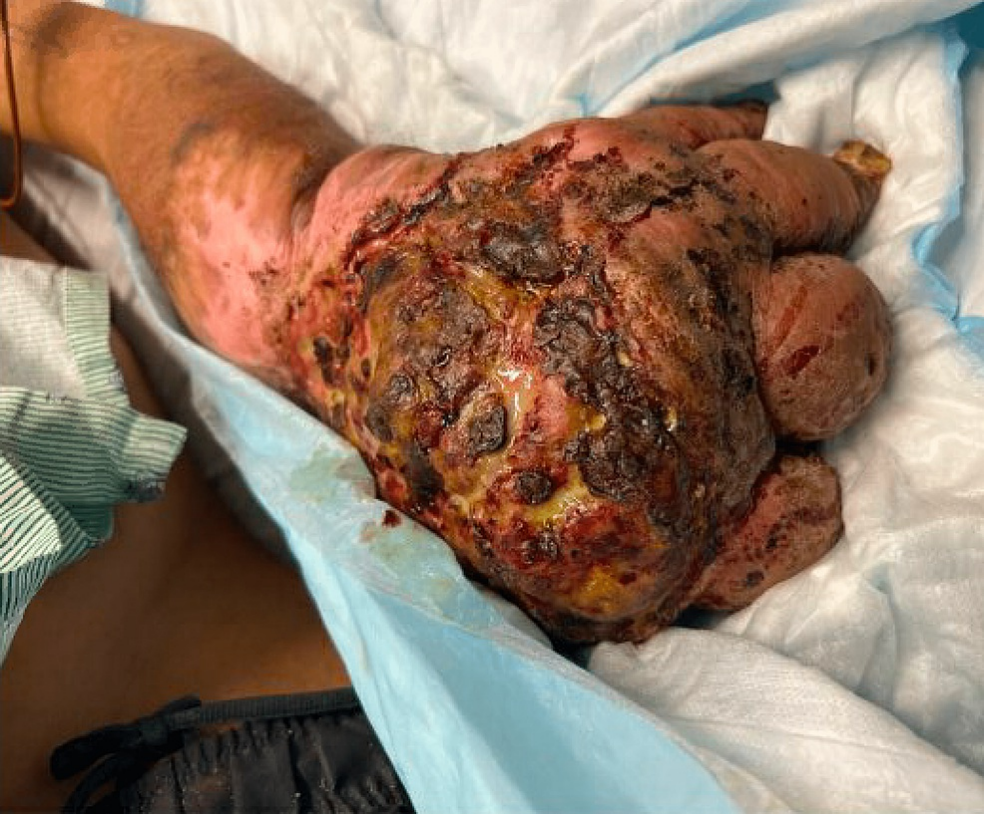
Representative image of the left hand demonstrating purulent discharge emanating from the center of the chronic wound overlying the dorsum of the hand.

On physical exam, he was afebrile with stable vital signs. The patient appeared chronically ill, and his left hand and forearm showed significant deformity, in addition to a chronic wound of the dorsum of the left hand and wrist draining purulent fluid, as shown in Figure 1. Labs were notable for Hb of 4.8 g/dL (MCV 69), WBC of 12.4 K/uL, platelets of 775 K/uL, creatinine of 0.64 mg/dL, and alkaline phosphatase of 251 U/L. Superficial cultures obtained demonstrated polymicrobial growth (group B Streptococcus and Proteus mirabilis) and, concerningly, heavy growth of Corynebacterium diphtheriae. Per infectious disease service recommendations, he received vancomycin initially and then, once susceptibility testing returned, penicillin G IV four million units every four hours to complete 10 days of treatment. He was also given trimethoprim-sulfamethoxazole double strength one tablet twice daily for surrounding cellulitis. Antitoxin therapy was deferred given isolated cutaneous disease. Given the lesion’s atypical appearance and concern for underlying malignancy, a biopsy was recommended; however, the patient left against medical advice prior to obtaining tissue. The organism was later confirmed to be C. diphtheriae var. mitis, and it was non-toxigenic by Elek testing at the CDC.

## Discussion

Interestingly, our patient’s wounds did not have the characteristic ulcerative appearance; rather, they appeared bulky and tumor-like, which prompted the recommendation to obtain a tissue biopsy. There was a concern for possible malignancy in the context of chronic inflammation. He, unfortunately, left against medical advice before a biopsy could be obtained for a definitive pathologic diagnosis.

Our patient reported that he had received all childhood vaccinations but was unsure if he had received any boosters as an adult. He likely had waning immunity in the setting of not obtaining a diphtheria booster (i.e. TDaP) in adulthood and was at increased risk due to frequent skin trauma from intravenous drug use without adequate hygiene. He was undomiciled and therefore did not have ready access to clean water to clean his wounds. He denied any travel history outside of the Midwest. 

It should be noted that vaccination against diphtheria generally confers protection against toxin-producing organisms but does not shield recipients from colonization or localized infection, as reported on the CDC's website [4]. If there is a concern for cutaneous disease, contact precautions should be maintained until the elimination of the organism is documented by two consecutive negative cultures once antibiotic therapy is completed. Of note, in the United States, confirmatory Elek testing is performed only by the CDC’s Pertussis and Diphtheria Laboratory.

Literature review yields that most cases of cutaneous diphtheria in the United States are contracted overseas in endemic regions, though there have been two major outbreaks in North America [2] that were thought to be locally contracted and spread. Our patient belonged to a similar demographic described in those outbreaks.

## Conclusions

Locally contracted diphtheria is rarely described in the United States due to the high prevalence of vaccination. While vaccination can help prevent toxigenic diphtheria, non-toxigenic diphtheria (especially cutaneous) can still cause localized infection, as described in this case. In addition, as the crisis of migrants deepens and the “anti-vax” movement remains strong in parts of the country, practitioners should be alert to previously rare and atypical presentations of vaccine-preventable illnesses.
